# Influence of personality traits on online self-disclosure: Considering perceived value and degree of authenticity separately as mediator and moderator

**DOI:** 10.3389/fpsyg.2022.958991

**Published:** 2022-08-17

**Authors:** Yuxiang Lv, Gege Fang, Xiaoxue Zhang, Yafei Wang, Yihuan Wang

**Affiliations:** ^1^School of Journalism and Communication, Tsinghua University, Beijing, China; ^2^Faculty of Arts and Social Sciences, National University of Singapore, Singapore, Singapore; ^3^School of Journalism and Communication, South China University of Technology, Guangzhou, China

**Keywords:** personality traits, perceived value, self-disclosure, social media, self-discrepancy theory

## Abstract

The Chinese have been spending significantly more time on the Internet during post-pandemic time. When people are confined indoors, there is a greater need to construct an identity and socialize online. Personality traits and perceived value of the media are important factors that affect users' online self-disclosure. This study involved the construction of a mediation model and a moderator model to evaluate the influence of personality traits on self-disclosure on social media. Perceived value was regarded as the mediator while the degree of authenticity was regarded as the moderator. Using a quota sampling method, 1,075 Chinese netizens were surveyed in July and August 2021. The results showed that: (1) The depth of self-disclosure of subjects with extroverted personality was significantly higher than that of those with introverted personality, that is, personality traits affect the depth of self-disclosure; (2) perceived value plays a mediating role in online self-disclosure; (3) The degree of authenticity is a significant moderator in the relationship between personality and self-disclosure. In all, the results from this study contribute to our understanding of how personality traits affect perceived value of media and self-disclosure. This study tested the credibility and validity of the proposed model in the context of the recent COVID-19 pandemic lockdown in China, and the study is a novel approach in that area of research.

## Introduction

The COVID-19 pandemic has profoundly changed people's lives and work. When people's offline movements are restricted, the experience of receiving and sharing information online has a greater impact on their psychological wellbeing. The pandemic has changed the way people interact socially. In this study, particular attention is paid to how trust is created and negotiated in online interactions. In a post-pandemic world where multiple risks coexist, trust promotes social interaction and shared vision as well as online self-disclosure (Niu and Meng, 2019; Li et al., 2022). While working from home, many people maintain interpersonal relationships both at work and within the family by self-disclosure online. In other words, they actively display and share information with others *via* social media platforms. The information could be text, image, audio, video, or geographic location. Some studies have demonstrated that social media are indeed effective and efficient in promoting the quality of friendship by assisting students to build on existing relationships and make new friends during crisis periods, such as during the COVID-19 pandemic lockdown (Chen, 2015; Wen et al., 2016; Amosun et al., 2021). Therefore, digital interactions supported by trust in the context of globalized risk should be one of the focuses of research in computer-mediated communication.

Self-disclosure refers to the voluntary and active self-information-sharing behavior of an individual (Wheeless and Grotz, 1976). It is a decision made by someone after comprehensively weighing potential benefits and risks, and it is influenced by several factors including social environment, psychological attachment, and personality traits (Binder et al., 2009). Self-disclosure is an important step for social media users in constructing their online identity and developing interpersonal relationships. However, the nature of the Internet makes the information disclosed by users to be shared with the world in real time (Lenhart and Madden, 2007), which leads to the risk of personal information misappropriation and abuse. Many researchers have explored the factors that affect the self-disclosure of social media users. Generally speaking, such factors can be categorized into five kinds: (i) Cost, that is, the perceived risk of self-disclosure, privacy value, etc. (Xu et al., 2013); (ii) Benefit: satisfying the motivation for entertainment, getting pleasure, etc. (Culnan, 2001); (iii) Personality: personality traits, etc. (Huang, 2016); (iv) Platform: function, incentive mechanism, etc. (Van Gool et al., 2015); and (v). Environment: cultural atmosphere, etc. (Posey et al., 2010). However, the research results in this field are relatively sparse.

Significant research attention has been paid to the impact of perceived risk of privacy and risk-benefit perception on self-disclosure on the Internet (Krasnova et al., 2010; Lu, 2019; Niu and Meng, 2019; Zhang and Li, 2019). In this study, close attention is paid to the influence of personality traits (introverted and extroverted personality) on self-disclosure behavior. The complexity of self-disclosure requires mixed research methods, but few empirical conclusions can be referred to. Most research have taken interpersonal trust on social network and perceived risk as mediators for model construction (Niu and Meng, 2019; Zhang and Li, 2019), whereas a few studies have considered the impact of users' self-disclosure on online identity construction and interpersonal relationship.

To sum up, this study explored the influence of personality traits on self-disclosure and focused on the relationship between the degree of authenticity in self-disclosure and the impact of personality traits on the depth of self-disclosure. The relationship between multiple variables was analyzed through mixed research methods. According to interpersonal relationship on social media and online identity construction, the study further explored the relationship among self-presentation, self-disclosure, perceived risk of privacy, and perceived value.

## Literature review

### Personality traits and the depth of self-disclosure

Individual behavior is influenced by personality traits. Early research on personality traits focused on vocabulary (Fiske, 1949; Norman, 1963; Tupes and Christal, 1992), which classified personality types through semantic interpretation and analysis. To measure personality traits of different individuals in a more convenient way, Zuckerman et al. (1993) designed the *Zuckerman's Personality Questionnaire* in 1994, which measured individuals' personality traits according to five factors. McCrae and Costa (1997) summarized five traits of individuals, namely, neuroticism, extraversion, openness, agreeableness, and conscientiousness. These five traits constituted the five-factor model of personality. Neuroticism is mainly manifested in the privacy anxiety of social media users during self-disclosure. Extraversion is related to social media users' social skills, self-confidence, and positive emotions. Agreeableness is related to social media users' trust and altruism during self-disclosure. Openness refers to the complexity and depth of the spiritual and experiential life of social media users. Conscientiousness is related to information control and the reliability of social media service providers (Wang et al., 2012).

Self-disclosure of social media users is affected mainly by extroversion and openness (Aharony, 2013). Rosengren (1974) argues that individual differences such as age, gender, and personality could affect people's use of mass media. Correa et al. (2010) examined the relationship between social media use and users' personality and found that people who were outgoing, emotionally stable, and open-minded tended to use the social media more frequently. In a study on the relationship between Facebook use and personality traits, Ross et al. (2009) found personality traits to be linked to several functions and motivations and extroverted people to be more likely to join Facebook groups. According to path analysis, Hollenbaugh and Ferris (2014) and Chen et al. (2016) found that extroverted people disclosed more accurate personal information. In an Australian study, Ryan and Xenos (2011) found that Facebook users were more likely to have extroverted and narcissistic personalities. Personality traits theory is widely adopted in behavioral and social sciences research. For example, investigating the influence of personality traits of employees could be helpful in allocating suitable jobs, reducing unsafe and insecure behaviors, improving personnel arrangements for important positions and positions prone to safety accidents, and complementing work performance management strategies (Amabile, 1983; George and Zhou, 2001).

This study aims to demonstrate that posting certain content on social media is self-disclosure behavior driven by personal will, and that different personality traits will affect the self-disclosure behavior of online social media users. Therefore, Hypothesis 1 is stated as follows:

**H1-1**: The depth of self-disclosure of online users with extroverted personality is significantly higher than that of users with introverted personality.

### Mediating effects of perceived usefulness, perceived hedonism, and perceived value

The concept of perceived value was initially applied in the fields of management and marketing. The function and utility experienced by customers in the process of consumption were termed as customer perceived value. Scholars have developed a scale of perceived value to study customer purchasing behavior and customer purchasing evaluation, reflecting the relationship between customers and merchandise (Zeithaml, 1988; Oliver and Swan, 1989; Sheth et al., 1991; Kantamneni and Coulson, 1996; Sweeney and Soutar, 2001). With the continuous development and maturity of social media, the functions of social media, such as posting comments and sending bullet screen comments, have changed the relationship between audiences and media from that of unidirectional communication to bidirectional interaction. The role of users has also changed from being passive audiences to being active participants. As can be seen, the relationship between the audience and social media has a common point with the relationship between customers and merchandise, since both audience and customers can actively choose suitable products, leading to interpreting perceived value of media from the perspective of communication (Zhang et al., 2021). The Measuring Perceived Value Scale (MPV Scale) was developed according to the perceived value of media, dividing perceived value into five factors: emotion and social status value (EMO), entertainment value (ENT), social networking value (INT), organizational communication value (ORG), and information value (INS).

Overall, research on the perceived value of media has two strands. The first involves taking the perceived value of media as an independent variable to explore its impact on factors such as media use, satisfaction, and privacy disclosure. Perceived usefulness was found to positively affect the willingness to disclose personal information (Hunt et al., 2012; Nie and Luo, 2013). Valkenburg and Peter (2007) found that 32% of the socially anxious adolescents in their study perceived online communication as more valuable for intimately self-disclosing about a wide variety of topics than doing so offline. Krasnova et al. (2010) pointed out that the main motivation for users to disclose personal information was to maintain and develop interpersonal relationships and be entertained. Users who attempted to develop and expand interpersonal relationships were more likely to post sensitive personal information on social media (Nosko et al., 2010). Oliver (2014) regards perceived hedonism as an additional psychological need of users. The hedonic value experienced by users is positively associated with users' satisfaction with social media applications. Applications based on image processing provide millions of special effects, filters, and stickers to enhance the beautification of images. Such functions not only satisfied users' perceived hedonism but also improved their satisfaction with the applications and services.

The second research strand involves exploring the composition of the perceived value of media. Scholars have developed different scales (based on extensive research) related to the perceived value of media, which have been widely used in communication and sociology research. Hirschman and Holbrook (1982) propose that perceived value not only includes utilitarian value (the balance between perceived gains and losses) but also includes symbolic, hedonic, and esthetic values. Holbrook (1999) further classifies perceived value into interaction, experience, and preference values according to three distinct dimensions, namely, the essence, orientation, and motivation of value. Li (2017) studied the customer behavior of e-book purchasers through four factors: emotional value, social value, price value, and quality value. Zhu et al. (2019) explain why audiences were addicted to short videos using four factors: social value, content value, interactive value, and entertainment values. Currently, the most widely adopted perceived value scale is composed of five factors: perceived usefulness, perceived hedonism, perceived ease of use, privacy risk, and perceived value.

In this study, it is proposed that using social media satisfies the requirements of users in the different dimensions, which affects the perceived value of social media. Therefore, with reference to the uses and gratifications theory, as well as the influence of perceived usefulness, perceived hedonism, and perceived value on self-disclosure, the following hypotheses are proposed:

**H2-1**: Extroverted personality affects perceived usefulness, which in turn affects the depth of self-disclosure.
**H2-2**: Extroverted personality affects perceived hedonism, which in turn affects the depth of self-disclosure.
**H2-3**: Extroverted personality affects perceived value, which in turn affects the depth of self-disclosure.

### Moderating effect of the degree of authenticity in self-presentation

The theory of self-presentation was first proposed by sociologist Goffman (1959) according to symbolic interactionism. Goffman (1959) suggested that life was like a stage, and that self-presentation was a performance on that stage. Self-presentation is defined as the goal-directed activity of controlling information to influence the impressions formed by an audience about the self (Schlenker and Wowra, 2003). When social media users edit personal data on social media platforms to construct virtual identities for online dialogue, personal data become a key tool for users to present themselves (Joinson, 2008). In cyberspace, the virtual and anonymous nature of identity offers users a certain space and freedom to edit their personal data. Users could do “selective exposure” by editing personal data, publishing media content, and conducting online conversations to improve their identity construction. Goffman (1956) conceptualize identity as an ongoing self-disclosure, arguing that the construction of identity was a process of “impression management” in which people habitually monitored audience feedback.

Hogan (2010) extended Goffman's dramaturgy theory, showing that the presentation of self in the age of social media was a continuous process, accompanied by the display of various exhibits (such as photos and short videos in WeChat Moments). Consequently, the construction of a user's identity has evolved from real-time improvisational performances to permanent online exhibitions, with social media accounts serving as exhibition halls for image-making practices of smartphone users (Carah, 2014). The content is preserved for longer periods and has greater impact on the user's personal image. Therefore, users tend to present improved images of themselves online. Some research works (Papacharissi, 2010; Guo and Yang, 2020) have demonstrated that “photoshopped” pictures used on social media conformed to the traditional concept of impression management.

In general, self-disclosure on social media is decorative. Users can create various “personalities” according to different requirements and can thus build several “virtual selves.” From this, we propose the core difference between self-presentation and self-disclosure thus: self-presentation can be decorative, whereas self-disclosure is based on truth, and self-disclosed information is more in line with people's real-life situations. However, self-disclosure and self-presentation are not mutually exclusive (Schlosser, 2020); both contribute to identity construction on social media platforms. This paper therefore adapts the construction of the Self-Disclosure Scale to accommodate the current situation of multimedia technology use and the prevalence of cosmetically modified and technically enhanced social performances.

In self-discrepancy theory, it is held that it is difficult for users to fully realize or express the ideal self, ought self, and actual self in the real world (Higgins, 1987). To meet various needs (such as hobby discovery, work requirements, and social networking), users' self-disclosure on social media is often accompanied by personality expression and identity construction at different levels (Seidman, 2014). Owing to the existence of such self-discrepancy, people are more willing to present or express the ideal self and ought self on social media. Such self-disclosure not only improves the efficiency of personal image management of users but also accelerates the self-beautification tendency of self-disclosure on social media. In a study of the relationship between self-disclosure strategies on the social media platforms of police officers and the satisfaction of the public, Wang et al. (2020) found a significant positive correlation between police officers' positive self-disclosure and positive emotions expressed by their clients on social media. Previous research has shown that moderate-extroverted users are more willing to present themselves than over-extroverted users (Bao, 2013). Nonetheless, extroverted users are more willing to post carefully beautified photos with unique styles (Krämer and Winter, 2008). Thus, although social media provides new channels and ways for users to present themselves, users' self-disclosure is not 100% free from constraints.

Self-disclosure acts are often subject to assessments of authenticity (Schlenker, 1975; Buss and Briggs, 1984; Tesser and Moore, 1986; Leary, 1993); if we reveal only our desirable qualities, we are showing only a very narrow “sample” of our true selves (Jiang et al., 2020). Such selective disclosure will affect the audience's trust in the content disclosed by users. Especially in the context of global risks, where most of people's production and life behaviors are exhibited online, online trust is particularly significant. The degree of authenticity in self-disclosure has an impact on the self-disclosure of social media users, and the exact degree is determined by numerous factors, such as users' real identity, vocation, and personality traits. Nevertheless, in the virtual world constructed on social media, external constraints are reduced. Users are able to publish social media content based purely on their own preferences, emotions, and value judgments, which creates a large gap between identity construction in the real world and that in the virtual world. Studies have shown that in a quasi-social interaction environment, intimate self-disclosure behavior can more realistically reflect users' daily lives; thus, a high degree of authenticity of a user's self-disclosure helps the audience to accept the information disclosed by the user (Nah, 2022).

While most scholars have focused on exploring the different factors that affect self-disclosure, little attention has been paid to the fact that the degree of authenticity in self-disclosure may interact with, or otherwise moderate, empirical research results. The effect of the truthful presence on self-disclosure has not received significant research attention. Accordingly, this study aims to show that in the context of post-pandemic global risks, the variable of authenticity cannot be ignored because online trust is an important factor in promoting the return of benign communication between social individuals and groups. Therefore, the degree of authenticity in self-disclosure has been introduced into this study as a moderator variable, and Hypothesis H3-1 is proposed as follows:

**H3-1**: Personality traits have an impact on the depth of self-disclosure, but this effect is positive when the degree of authenticity in self-disclosure is stronger, and vice versa.

Perceived risk is an important factor affecting users' self-disclosure on social media. It refers to people's perception of potential losses they might encounter when chasing ideal results (Featherman and Pavlou, 2003). Derlega and Chaikin (1977) re-conceptualized self-disclosure as a form of boundary adjustment in the maintenance of privacy. Privacy breaches have also been cited as one of the reasons for the reduced willingness to self-disclose (Xu et al., 2013; Green et al., 2016). The traditional sense of Internet privacy perception refers to users' perceptions of the possibility that Internet service providers would protect their personal confidential information from improper use or disclosure (Kim et al., 2008). Many quantitative studies have shown that there is no significant correlation between perceived online privacy risk and self-disclosure (Taddei and Contena, 2013; Niu and Meng, 2019).

“Privacy paradox,” as proposed by Barnes (2006) in 2006, holds that people do not realize the public nature of the Internet. On the one hand, they worry about the invasion of privacy. On the other hand, they disclose personal information actively. Bazarova (2012) proposed another explanation: even if people knew that self-disclosure had privacy risks, they would still share selective personal information in order to establish and maintain an intimate relationship on the Internet. The privacy paradox, that is, the dichotomy between how a person intends to protect their online privacy vs. how they actually behave online (and how they do not protect their information online), has necessitated scholars to explore the factors that affect the personal expression and self-disclosure of social media users. The research results illustrate two influences: positive promotion and negative inhibition. Some studies have demonstrated that trust can promote users' self-disclosure. Users would be more willing to disclose personal information if they had a higher degree of trust in social media (Chen, 2010; Colomo et al., 2010; Lo and Riemenschneider, 2010; Salleh et al., 2011). Accordingly, a higher degree of trust in social media would contribute to in-depth self-disclosure by users (Niu and Meng, 2019). In contrast, in a study on personal privacy and security of college students' use of social media, Zhang and Li (2019) found that college students' perceived risk of social media (such as the belief that publishing information on social networking sites is unsafe, and that the published information may lead to the threat of privacy leakage) would inhibit the degree of self-disclosure on social media.

The Chinese consider interpersonal relationships to be very important (Chan, 2006; Buckley et al., 2010). Therefore, the contradictions of the privacy paradox are even more significant. The Chinese are concerned about the risks associated with sharing information on social media. Yet, social media have become instrumental to their personal and professional wellbeing (Huang and Miao, 2020). This study aims to show that there are many privacy factors that affect people's self-disclosure behavior, including the balance between risk and return, personality, psychological state, and position. However, the nature of the correlation remains unclear, given the possible mediating effects of different factors. Considering this, this study raises the following hypotheses:

**H4-1**: There are different factors that motivate users' expressions and sharing on Weibo.
**H4-2**: There are different factors that inhibit users' expressions and sharing on Weibo.

Previous studies have found that a number of factors influence users' willingness to self-disclose on social networking sites, ranging from personality traits and interpersonal trust, to perceived social benefits and privacy risks (Abramova et al., 2017). These antecedents provide evidence for the complex nature of self-disclosure and the need for further research in this field.

**Figure 1:**
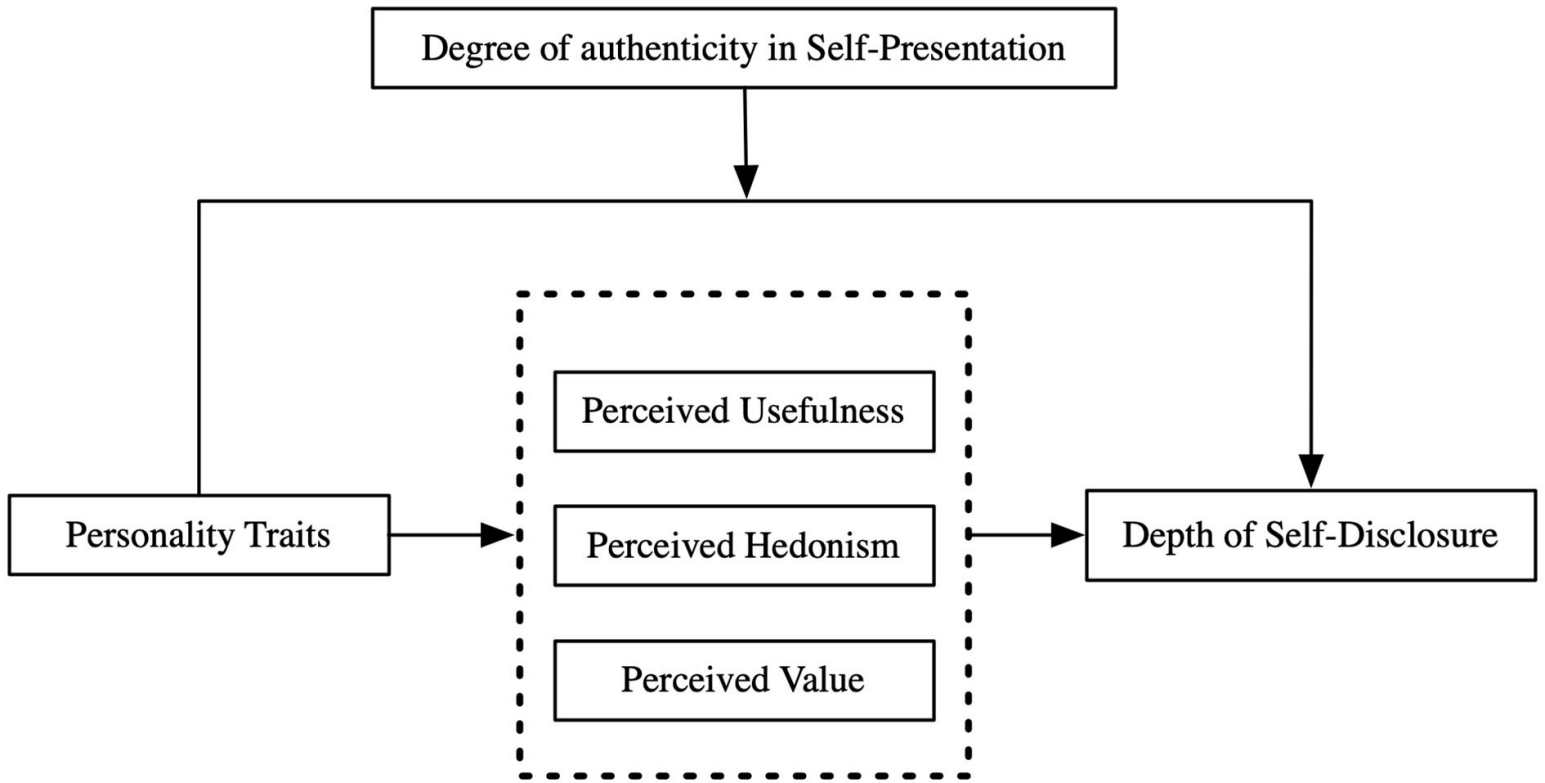
The research model.

According to the above discussion, Figure 1 illustrates the research model of the study.

## Methodology

### Respondents and sampling methods

Quantitative research methods were chosen according to the systematic and comparative analysis of the literature about the reciprocal relations among personality, perceived value, and self-disclosure. The quota for sampling was designed according to the 48th Statistical Reports on Internet Development in China released by China Internet Network Information Center, as well as the demographic statistics of China released by the National Bureau of Statistics at the end of 2020. The dimensions of quota sampling used were gender, age, region, educational level, monthly income, and place of residence. For gender, the male-to-female ratio was 5.5:4.5. For age, the bands were 19 years old and below, 20–29 years old, 30–39 years old, and 40 years old and above; the ratios of these four age groups were 1.8:2.7:2.5:3. Four regions were identified: east, central, west, and northeast; the ratios of these four regions were 3.8:2.7:2.7:0.8. Three educational levels were identified: high school and below, junior college, and undergraduate and above, with the ratios being 8:1.1:0.9. Four monthly income segments were identified as follows: below 3,000 yuan, 3,001–5,000 yuan, 5,001–8,000 yuan, and above 8,001 yuan; the ratios of these four income groups were 5.1:2:1.4:1.5. Four kinds of place of residence were identified: prefecture-level city, county-level city, township, and countryside, with the ratios of these four places of residence being 4.9:4.1:0.5:0.5.

### Questionnaire design

The questionnaire contained three scales that are widely regarded as mature scale by most researchers, namely, Personality Traits, Perceived Value of Media, and Depth of Self-Disclosure. Question design for the independent variable: personality traits relied mainly on the *Eysenck Personality Questionnaire-Revised, Short-Scale for Chinese* (EPQ-RSC), which was revised by Qian et al. (2000) in 2000. It has been verified to be reliable and valid; it also meets the requirements of psychometrics and is suitable for Chinese respondents. The overall scale is divided into four subscales, among which is the Extroversion Scale (E), which is used to distinguish between extroverted and introverted personalities. Thus, the variable “personality traits” is binary. According to the scoring of the questions, respondents who score 8 points or more are assigned to the extroverted personality category, while those who score <8 are assigned to the introverted personality category.

The Scale of Perceived Value of Media was adapted from the Perceived Value Scale (Voss et al., 2003; Kim et al., 2007; Deng et al., 2021), which lists three factors: perceived usefulness, perceived hedonism, and perceived value. Perceived usefulness was measured by asking the degree of agreement with statements like “Posting original posts on Weibo is valuable to me,” “Posting original posts on Weibo helps me solve health problems,” and “Posting original posts on Weibo helps me accumulate social connections” (adapted from Deng et al., 2021). Perceived hedonism was measured by asking the degree of agreement with statements like “I enjoy the process of posting original posts on Weibo,” “Posting original posts on Weibo makes me happy,” and “Posting original posts on Weibo is very interesting” (adapted from Voss et al., 2003). Perceived value was measured by asking the degree of agreement with statements like “Posting updates on Weibo is beneficial to me compared with the required effort,” “Posting updates on Weibo is worthwhile compared with the required time and effort,” and “Sharing my life on Weibo is meaningful for me despite privacy risk” (adapted from Kim et al., 2007). As WeChat has more than 1.2 billion monthly active users in China, it is recognized as an infrastructure-based platform with a low threshold for usage skills. Therefore, we eliminated the “perceived ease of use” from the original scale, since Weibo is intended to be an easy-to-use platform designed for the popularization of new technologies and design. Subsequent sections were designed to separately measure how the three sub-dimensions played mediating roles in the relationship between personality traits and the depth of self-disclosure. The final section of the questionnaire was designed to measure the degree of authenticity in self-disclosure. With self-assessment questions, this final part focused on the gap between the online persona and the real person of the respondents.

Finally, we paid close attention to optimizing the scale of self-disclosure. Currently, the measurement of self-disclosure in the research mainly focuses on three aspects: depth, breadth, and quantity (Posey et al., 2010; Huang, 2016). Considering the access to multimedia technologies on social media, this study included options on forms of sharing to measure the depth of self-disclosure (video > image > text).

The full version of the questionnaire is available online at https://www.frontiersin.org/articles/10.3389/fpsyg.2022.958991/full#supplementary-material.

To ensure the reliability of the revised scale, a pilot test was conducted with 26 users. The statistical results of the pilot showed that the revised scale was reliable and valid, and thus could be used for the research.

### Data collection

The questionnaire was made available online between July 30 and August 15, 2021. A total of 1,196 responses were collected. The average time to fill in the questionnaire was about 3 min. Three types of invalid responses were excluded: if the questionnaire was completed in <60 s (1 min), if the questionnaire was completed in more than 1,000 s (16.67 min), and where there were inconsistent responses. After exclusion, the number of valid questionnaires was 1,075 (refer Table 1).

**Table 1:** Descriptive statistical analysis of sample structure (*N* = 1,075).

		**Frequency**	**Percentage**	**Cumulative percentage**
Gender	Male	662	61.58	61.58
	Female	413	38.42	100.00
Age	24 years old and below	390	36.28	36.28
	25–34 years old	553	51.44	87.72
	35–44 years old	96	8.93	96.65
	45 years old and above	36	3.35	100.0
Education	Primary school and below	17	1.58	1.58
	Junior high school	67	6.23	7.81
	Senior high school; technical secondary school; vocational/occupational training school	235	21.86	29.67
	Junior college	273	25.40	55.07
	Undergraduate	436	40.56	95.63
	Master and above	47	4.37	100.0
Place of residence	Prefecture-level city	532	49.49	49.49
	County-level city	439	40.84	90.33
	Township	69	6.42	96.75
	Countryside	35	3.26	100.0
Total		1,075	100.0%	100.0%

The effective recovery rate was 89%, and the effective sample details in the dataset can be accessed *via* URL https://www.frontiersin.org/articles/10.3389/fpsyg.2022.958991/full#supplementary-material.

The majority of the respondents were male (61.58%) (female: 38.42%); ages below 34 accounted for 87.72% of all respondents. Undergraduates accounted for 40.56% of the total sample size. In terms of place of residence, most respondents came from prefecture-level (49.49%) and county-level (40.84%) cities. The difference between the final sample and the sampling target (actual target) was between plus or minus 0.1–7%. Therefore, the final sample was representative and could reflect the overall situation and trend of the Chinese population of Internet users.

### Data analysis

SPSS 26.0 was used for the statistical analysis. More precisely, the data were analyzed using descriptive statistical means; normality tests of the collected data were carried out, and the reliability and validity of the scales were tested. Considering multiple responses, significant differences were detected between factors that motivate users to express and share their lives on Weibo and those that inhibit them from doing so. Spearman's rank correlation analysis was used to calculate the relationship between the different variables. Moderating effect analysis was conducted to measure the moderating effect of the variable, the degree of authenticity in presentation, on both personality traits and the depth of self-disclosure. The plug-in function of SPSS 26.0, Process, was used to test the mediating effects of the mediating variables, namely, perceived usefulness, perceived hedonism, and perceived value. The test level was α = 0.05, and *p* < 0.05 was considered statistically significant.

## Research results

### Descriptive statistical analysis

Among the many methods of self-presentation on the Internet, sharing hobbies, book lists and movie reviews, and professional and industrial knowledge were the commonest, with 446 (15.1%), 443 (15.0%), and 413 (14.0%) users, respectively. In comparison, only 50 users (1.7%) shared their emotions and insights. The differences in self-presentation online were statistically significant (χ^2^ = 624.68, df = 9, *p* < 0.001).

“To manage one's social relations (500 respondents, 46.5%)” and “to facilitate one's work (500 respondents, 46.5%)” were the two main factors that motivated online sharing behavior. In contrast, only 50 persons (1.7%) shared their emotions and insights, making it a relatively insignificant factor. The motivation of the self-disclosure behavior of respondents was statistically significant (χ^2^ = 451.86, df = 7, *p* < 0.001), which establishes Hypothesis H4-1.

Among the factors that inhibit sharing (refer Table 2), “worry about the pressure from public opinion (482 respondents),” “worry about the pressure from social relations (460 respondents),” and “worry about privacy leak (460 respondents)” were the three most important. In contrast, only 76 respondents (2.8%) believed that sharing frequently online was naive. Factors that inhibit sharing were statistically significant (χ^2^ = 483.95, df = 7, *p* < 0.001), which establishes Hypothesis H4-2.

**Table 2:** Factors that inhibit sharing (*N* = 1,075).

**Factors that inhibit sharing**	**Responses**		**Percentage of individual cases**
	***N***	**Percentage**	
Worry about privacy leak	460	16.8%	42.8%
Less feedback from friends	418	15.3%	38.9%
Worry about the pressure from public opinions	482	17.6%	44.8%
Worry about the pressure from social relations	460	16.8%	42.8%
Lack of interesting topic	384	14.1%	35.7%
Lack the desire to share	304	11.1%	28.3%
The frequency of using social media is low	147	5.4%	13.7%
Sharing too frequently online is naive	76	2.8%	7.1%
Total	2,731	100.0%	254.0%

### Tests of normality, reliability, and validity

#### Test of normality

The result of the normality test is presented in Table 3. According to West et al. (1995) and Kim (2013), when the absolute value of skewness was <2 and the absolute value of kurtosis (−3) was <7, the data could be considered approximately normally distributed. All the above variables conformed to a normal distribution.

**Table 3:** A description of the normality of statistics (*N* = 1,075).

	**Mean**	**Standard deviation**	**Skewness**		**Kurtosis**	
			**Statistic**	**Standard error**	**Statistic**	**Standard error**
Perceived usefulness	4.0258	0.80297	−0.832	0.075	0.835	0.149
Perceived hedonism	4.0016	0.83559	−0.908	0.075	1.073	0.149
Perceived value	3.9953	0.84347	−0.997	0.075	1.298	0.149
The depth of self-exposure	3.9326	0.78426	−0.650	0.075	0.524	0.149
The degree of authenticity in self-presentation	4.0685	0.80293	−0.818	0.075	0.591	0.149

#### Test of reliability and validity

Reliability is tested by comparing the results produced when repeatedly measuring the same object with the same methods. The reliability coefficient was positively correlated with the consistency of the results, and thus established the validity and stability. Additionally, this study tested the scales in the questionnaire through construct validity. Maximum rotation of variance was adopted to test the collected data.

Table 4 shows that the result of Cronbach's Alpha test was 0.967 (>0.8), reflecting that the results of this study are reliable. The results of Cronbach's Alpha test of perceived usefulness, perceived hedonism, perceived value, the degree of authenticity in self-presentation, and the depth of self-disclosure were above 0.8, indicating a high degree of reliability. In other words, all the variables in the questionnaire passed the reliability test. (The personality trait was a binary variable for which no test was needed.) Table 5 shows the KMO value was 0.981 (>0.8) and presents the results of the Bartlett test of sphericity (*p* = 0.000 < 0.05). In other words, the validity of the research data was also established.

**Table 4:** Result of Cronbach's alpha test.

**Variable**	**Cronbach's alpha**	**Number of items**
Perceived usefulness	0.875	4
Perceived hedonism	0.845	3
Perceived value	0.849	3
The degree of authenticity in self-presentation	0.861	3
The depth of self-disclosure	0.917	8
Total	0.967	21

**Table 5:** KMO and Bartlett test.

**Kaiser-Meyer-Olkin measure of sampling adequacy**		**0.981**
Bartlett test of sphericity	Approximate Chi-Square	17,466.476
	Approximate Chi-Square df	210
	Sig.	0.000

### Correlation analysis

When dealing with quantitative data, correlation analysis is a widely accepted statistical method for studying the relationships among variables. It is useful in determining whether a relationship exists among variables, and if so, to what degree. This study adopted the Spearman rank correlation analysis (refer Table 6 for details).

**Table 6:** Spearman rank correlation analysis on the variables.

**Correlation analysis**	**Perceived usefulness**	**Perceived hedonism**	**Perceived value**	**The degree of authenticity in self-presentation**	**The depth of self-disclosure**
Perceived usefulness	1				
Perceived hedonism	0.869^**^	1			
Perceived value	0.829^**^	0.848^**^	1		
The degree of authenticity in self-presentation	0.798^**^	0.769^**^	0.777^**^	1	
The depth of self-disclosure	0.824^**^	0.809^**^	0.817^**^	0.827^**^	1

The correlation was significant at a confidence level of 0.01 (two-tailed test).

** p < 0.01.

Table 6 shows that perceived usefulness, perceived hedonism, perceived value, the degree of authenticity in self-presentation, and the depth of self-disclosure are all significant factors in online self-disclosure, with the correlation coefficient values being 0.824, 0.809, 0.817, and 0.827, respectively, and the correlation coefficient values being >0. This indicates that the depth of self-disclosure and perceived usefulness (*r* = 0.824, *p* < 0.01), perceived hedonism (*r* = 0.809, *p* < 0.01), perceived value (*r* = 0.817, *p* < 0.01), and the degree of authenticity in self-presentation (*r* = 0.827, *p* < 0.01) are all positively correlated. The significance was at a confidence level of 0.01.

### *T*-test of independent and dependent variables

To conduct the *t*-test, this study conceptualized the independent variable personality trait as a binary variable (extroverted and introverted personalities), and the dependent variable depth of self-disclosure as a continuous variable. The test results are presented in Table 7.

**Table 7:** Group statistics of different personality traits.

	**Personality traits**	**N**	**Mean**	**Standard deviation**	**Standard error of mean**
The depth of self-disclosure	Extroverted personality (scored 8 or higher)	813	4.0617	0.7376	0.0259
	Introverted personality (scored lower than 8)	262	3.5320	0.7909	0.0489

Shapiro–Wilk Normality Test was used to test the normality of the depth of self-disclosure (grouped by extroverted and introverted personalities). The results showed that the depth of self-disclosure was normally distributed in both groups, which was consistent with the *t*-test requirements. The homogeneity of variance test showed that the variances of the two groups of data were equal (*F* = 0.316, *p* = 0.574 > 0.05), and the *t*-test result was *t*_(df)_ = 9.93 (1,073), *p* = 0.000 < 0.05 (refer Table 8). Therefore, the depth of self-disclosure for both the extroverted personality (4.06 ± 0.74) and the introverted personality (3.53 ± 0.79) was statistically significant. However, the depth of self-disclosure of extroverted personality was significantly higher than that of introverted personality, establishing Hypothesis H1-1.

**Table 8:** *T*-test of independent samples.

		**Levene test of the variance equation**		***T*** **-test of the mean equation**				
		**F**	**Sig**.	**t**	**df**	**Sig**.	**The mean difference**	**Standard error difference**
The depth of self-disclosure	Assuming that variations are equal	0.316	0.574	9.93	1,073	0.000^***^	0.5297	0.0533
	Assuming that variations are not equal			9.58	417.238	0.000^***^	0.5297	0.05530

Two-tailed test,

*** < 0.001.

### Test of moderating effect

The test of moderating effect was performed to determine whether the degree of authenticity in self-presentation, as the moderating variable, affected the relationship between the independent variable (personality traits) and the dependent variable (the depth of self-disclosure) under different circumstances. Pre-processing of data is required before undertaking a moderating effect analysis. This study adopted the centralized processing method. The mean values of the variables of each group were first calculated. Then, the values of the variables of each group were deducted from the mean value of the group to get the desired values. Finally, the desired values were multiplied to sort out the interaction terms of each variable to observe the changes in the *P*-value and the R-squared.

Table 9 presents the moderating effect of using two models. Model 1 included the independent variable (personality trait) and the moderating variable (the degree of authenticity in self-presentation). In Model 2, the interaction terms were inserted between the independent variable and moderator variable according to Model 1. The purpose of Model 1 was to study the influence of the independent variable (personality traits) on the dependent variable (the depth of self-disclosure) without considering the interference of the moderating variable (the degree of authenticity in self-presentation). The results from Model 2 showed that the interaction between personality traits and the depth of self-disclosure was significant (*t* = 3.352, *p* = 0.001 < 0.05). The results demonstrate that the influence of personality traits on the depth of self-disclosure was significantly moderated by the moderating variable, the degree of authenticity in self-presentation.

**Table 9:** Moderating effect of the degree of authenticity in self-presentation (*N* = 1,075).

	**Model 1**				**Model 2**			
	**B**	**Se**	***t***	***p***	**B**	**Se**	***t***	***p***
Constant	3.933	0.013	295.747	0.000^**^	3.921	0.014	286.394	0.000^**^
Personality trait	0.129	0.032	4.024	0.000^**^	0.159	0.033	4.785	0.000^**^
The degree of authenticity in self-presentation	0.791	0.017	45.945	0.000^**^	0.797	0.017	46.250	0.000^**^
Personality trait × the degree of authenticity in self-presentation					0.126	0.038	3.352	0.001^**^
R^2^	0.691				0.694			

Dependent variable: the depth of self-disclosure, se: standard error,

** p < 0.01.

Taking personality traits as independent variables, the depth of self-disclosure as a dependent variable, and the degree of authenticity in self-presentation as the moderator variable, the moderating effect analysis showed that the degree of authenticity in self-presentation had a positive moderating effect on personality traits (b = 0.126, *p* < 0.01, ΔR2 = 0.003). When the degree of authenticity in self-presentation was high, personality traits had a strong positive impact on the depth of self-disclosure; when the degree of authenticity in self-presentation was low, personality traits still had a strong positive effect on the depth of self-disclosure, though the effect was smaller. These results confirmed Hypothesis H3-1.

### Test of mediating effect

Drawing on the test of mediating effect proposed by Preacher and Hayes (2008), the plug-in function of SPSS 26.0, Model 5, was used, where personality traits were taken as independent variables (introverted personality was used as the control group for reference), the depth of self-disclosure was taken as the dependent variable, and perceived usefulness, perceived hedonism, and perceived value were taken as mediating variables. The results in Table 11 were obtained *via* 5,000 samplings (at a 95% confidence interval).

As Table 10 shows, extroverted personality (SE = 0.0284, at 95% confidence interval: [0.0309, 0.1417]) had a significant indirect effect on the depth of self-disclosure through perceived usefulness. Extroverted personality (SE = 0.0335, 95% confidence interval: [0.0414, 0.1718]) had a significant indirect effect on the depth of self-disclosure through perceived hedonism. Extroverted personality (SE = 0.0274, 95% confidence interval: [0.0569, 0.1644]) had a significant indirect effect on the depth of self-disclosure through perceived value. In other words, perceived usefulness, perceived hedonism, and perceived value have mediating effects between personality traits and the depth of self-disclosure. Therefore, Hypotheses H2-1, H2-2, and H2-3 are established.

**Table 10:** Test of mediating effect (taking introverted personality as the control group for reference).

**Mediating path**	**Boot SE**	**Effect**	**95% BootLLCI-BootULCI**	**Conclusion**
Extroverted personality – perceived usefulness – the depth of self-disclosure	0.0284	0.0820	[0.0309, 0.1417]	Significant
Extroverted personality – perceived hedonism – the depth of self-disclosure	0.0335	0.1040	[0.0414, 0.1718]	Significant
Extroverted personality – perceived value – the depth of self-disclosure	0.0274	0.1067	[0.0569, 0.1644]	Significant

BootLLCI refers to the lower limit of the 95% interval of the Bootstrap sampling, and BootULCI refers to the upper limit of the 95% interval of the Bootstrap sampling. The 95% confidence interval of the mediation effect (ind_eff) was obtained via the bootstrap method. The statistical results are insignificant if the confidence interval contains 0 (the upper and lower limits of the interval have contrary signs). Otherwise, statistical results are significant.

## Conclusions and discussions

The COVID-19 pandemic has had an impact on people of all ages and “is giving us all a generational experience, debunking the fragility of our stable existence” (Fiedler, 2020). People with different personalities faced more complex and frequent online communication. By combining the influences of personality, perceived value, and authentic degree, this study provides an integrative framework for understanding the mechanisms behind self-disclosure behavior, providing reference for online management of companies and personal online presence. The results of the tests of hypotheses are presented in Table 11.

**Table 11:** Test results of hypotheses.

**Research hypothesis**	**Results**
H1-1: The depth of self-disclosure of extroverted personality is significantly higher than that of introverted personality.	Supported
H2-1: Extroverted personality affects perceived usefulness, which in turn affects the depth of self-disclosure.	Supported
H2-2: Extroverted personality affects perceived hedonism, which in turn affects the depth of self-disclosure.	Supported
H2-3: Extroverted personality affects perceived value, which in turn affects the depth of self-disclosure.	Supported
H3-1: Personality traits have an impact on the depth of self-disclosure, but this effect is positive when the degree of authenticity in self-presentation is stronger, and vice versa.	Supported
H4-1: There are differences among the factors that motivate users' expressions and sharing on Weibo.	Supported
H4-2: There are differences among the factors that inhibit users' expressions and sharing on Weibo.	Supported

First, this study confirms a positive correlation between extroverted personality and perceived value of media, as well as a positive correlation between extroverted personality and social media self-disclosure. It further establishes the moderating effect of the degree of authenticity in self-presentation on the relationship between personality traits and self-disclosure. In general, extroverted users are more likely to recognize the usefulness, hedonism, and the accumulated value of social capital while using social media. Consequently, these users tend to display deeper, wider, and more frequent self-disclosure behavior to communicate on social media.

Second, the degree of authenticity in self-presentation, which exhibits users' subjective cognition, exerts an influence on the correlation between personality traits and self-disclosure. When the degree of authenticity in self-presentation is high, the correlation will be positive, whereas when the degree of authenticity in self-presentation is low, the correlation will be negative. In contemporary society, self-beautification for self-disclosure on social media has been confirmed by numerous studies (Boyle and Johnson, 2010; Papacharissi, 2010; Qiu, 2021). Briefly, such behavior has penetrated daily contexts. Users will edit and beautify their posts on social media to different degrees (Papacharissi, 2010). They consciously and selectively display good moral qualities, insightful attitudes, and good-looking bodies when posting short videos (Qiu, 2021).

Users who are eager to communicate and want to establish serious relationships tend to show their most perfect selves on social media (Boyle and Johnson, 2010). Therefore, extroverted users equate authentic self-presentation with deep disclosure. They not only pursue deeper and wider self-disclosure, but also exhibit more authentic self-disclosure practices. In other words, they desire that the online image they project will become (closer to) their reality. Authenticity becomes the valuable foundation of all privacy sharing. A low degree of authenticity in self-presentation leaves little room for the growth of self-disclosure. Studies have shown that the anonymization of the web has created room for identity reinvention; yet, there is no data to show that identity fraud is more prevalent online. On the contrary, some studies have suggested that the anonymity and reduction of associated social risks may make people more honest in online self-disclosure than offline (McKenna, 2000).

The reduction of cues in computer-mediated communication does not take people further away from their real identities, but it sometimes allows them to express themselves more authentically. Because digital platforms have the potential to reshape the ecology of online communication and the new order of information dissemination, one of the improvements should be rethinking the value of authenticity in the online world. One way to improve the quality of people's online communication could be to improve the authenticity of online information, thereby increasing users' trust and perception of social media value. China has now implemented the mandatory display of IP addresses to provide Internet users with more reliable means of identifying creators and sharers of online content. Authentic self-disclosure can enhance the virtual social capital of netizens, promote information exchange and opportunity sharing, and help rebuild the shared vision of society in the post-pandemic world, while trust is an effective factor that improves the willingness of network members to share. As a result, enhancing trust could be considered as the core of any strategy to motivate knowledge sharing on social media.

Third, this study reveals the new trends created by the application of multimedia technology on social media. Audio and video communication forms, such as vlogs, live pictures, and live broadcasts, allow users to share their private life more realistically. As a way of attracting Internet traffic, multimedia content is widely used to shape the personal brands of Internet celebrities and marketing activities of e-commerce companies. As the findings above show, perception value plays a mediating role on the willingness of people to engage in self-disclosure online. Now, with the assistance of complex and rich technical affordances, users are provided with more choices and considerations for online self-disclosure. As there is room for growth in the perceived value enhancement, the online self-disclosure of future users may be more deeply connected to real life.

Furthermore, self-disclosure is closely related to self-presentation, image showcasing, and identity construction. Therefore, one of the innovations of this study is the incorporation of the dimension of identity construction into the self-disclosure scale. In addition to highlighting self-disclosure as a way of gaining support, this study also showed that self-disclosure is an important strategy for managing personal image. Furthermore, this study found that extroverted users were more active in the management and maintenance of personal image on social media, which, accordingly, brought more social capital and greater perceived value of media to these users.

Finally, this study established that the factors that motivate or inhibit users' self-disclosure on Weibo are diverse. Occupation, age, gender, profession, etc. may affect users' judgments and choices. More work is still required in the areas of social media self-disclosure and image management. One limitation of the study is that it did not focus on the proportion and causality of the influencing factors. As the connections between virtual space and real society are becoming increasingly close, future research is required to investigate the influence of other factors, such as socializing, occupational needs, and privacy protection on the degree of self-disclosure of Internet users.

## Data availability statement

The datasets presented in this study can be found in online repositories. The names of the repository/repositories and accession number(s) can be found in the article/Supplementary material.

## Ethics statement

Ethical review and approval was not required for the study on human participants in accordance with the local legislation and institutional requirements. Written informed consent from the patients/ participants or patients/participants legal guardian/next of kin was not required to participate in this study in accordance with the national legislation and the institutional requirements.

## Author contributions

YL, GF, and XZ contributed to conception and design of the study. GF and YiW collected the questionnaire data and organized the database. XZ and YaW performed the statistical analysis. YL and GF wrote the first draft of the manuscript. XZ, YaW, and YiW wrote the sections of the manuscript. All authors contributed to manuscript revision, read, and approved the submitted version.

## Funding

This work was supported by the National Social Science Fund of China under Grant (Number: 16BXW088).

## Conflict of interest

The authors declare that the research was conducted in the absence of any commercial or financial relationships that could be construed as a potential conflict of interest.

## Publisher's note

All claims expressed in this article are solely those of the authors and do not necessarily represent those of their affiliated organizations, or those of the publisher, the editors and the reviewers. Any product that may be evaluated in this article, or claim that may be made by its manufacturer, is not guaranteed or endorsed by the publisher.
