# Supplementary material for: Influence of personality traits on online self-disclosure: Considering perceived value and degree of authenticity separately as mediator and moderator
Source: Front Psychol. 2022 Aug 17;13:958991. doi: 10.3389/fpsyg.2022.958991 (PMC9428552; doi:10.3389/fpsyg.2022.958991)
Supplement: Supplementary file 1 [file Data_Sheet_1.PDF]

Investigations on Weibo Use and Online Identity

*The full version of this questionnaire has 22 questions, and 18 questions related to this research are included here.

Hello, we are a research team from the School of Journalism and Communication, Tsinghua University, doing a study on Weibo usage and online identity. The following questionnaire mainly wants to know your related usage habits and influencing factors when using Weibo (Sina Weibo). All your answers will be kept strictly confidential and will be used to analyze relevant academic questions after removing personal information.

Q1. How often do you share the following categories of information on Weibo?

	**Never**			**Very Frequently**	
News	1	2	3	4	5
Work, Study	1	2	3	4	5
Travel, Daily	1	2	3	4	5
Family, Friends, Pets	1	2	3	4	5
Mood, Feeling	1	2	3	4	5
Hobby	1	2	3	4	5
Help Information	1	2	3	4	5

Q2. Is the following situation consistent with your actual situation and opinion?

* 【Judgment question】Yes=1, No=0

	**Yes**	**No**
Are you a talkative person?	1	0
Are you a lively person?	1	0
Would you like to meet strangers?	1	0
Can you let yourself go and have fun at a lively party?	1	0

Are you often proactive when making new friends?	1	0
Can you easily breathe life into a dull party?	1	0
Do you enjoy being around people?	1	0
Do you like having a lot of buzz and excitement around you?	1	0
Do you like to tell jokes and talk about funny things?	1	0
Are you always full of energy in the eyes of others?	1	0
Can you make a party go smoothly?	1	0
Do you tend to stay inconspicuous in social situations?	1	0

Q3. To what extent do you agree with the following statement?

* [Matrix scale question]

	**Absolutely Disagre**				**Absolutely Agree**
Posting original updates on Weibo is valuable to me.	1	2	3	4	5
Posting original updates on Weibo helped solve my problem.	1	2	3	4	5
Posting original updates on Weibo helps my social resources.	1	2	3	4	5
Posting original updates on Weibo helps others understand me.	1	2	3	4	5

Q4. To what extent do you agree with the following statement?

* [Matrix Questions]

	**Absolutely Disagre**				**Absolutely Agree**
I enjoy the process of posting original updates on Weibo.	1	2	3	4	5
Posting original updates on Weibo makes me happy.	1	2	3	4	5

Posting original updates on Weibo is very interesting.	1	2	3	4	5

Q5. To what extent do you agree with the following statement?

* [Matrix scale question]

	**Absolutely Disagre**			**Absolutely Agree**	
Publishing original content on Weibo will cause my personal information to leak.	1	2	3	4	5
My personal information in Weibo may be collected, tracked and analyzed.	1	2	3	4	5
My private information in Weibo may be misused, inappropriately shared or sold.	1	2	3	4	5

Q6. To what extent do you agree with the following statement?

* [Matrix scale question]

	**Absolutely Disagre**			**Absolutely Agree**	
Compared with the effort I put in, posting original updates on Weibo is beneficial to me.	1	2	3	4	5
Compared to the time and effort it takes, posting original tweets is worth it to me.	1	2	3	4	5
Posting original tweets is still worth it to me despite privacy risks.	1	2	3	4	5

Q7. Factors that inhibit you from expressing and sharing your life on Weibo include:

* [Multiple choice questions]

Inhibiting Factors<br>Tick	√
Worry about privacy leaks	
less feedback from friends	
fear of public pressure	
Worry about social relationship pressure	
Lack of interesting topics	
lack of sharing	
less use of social media	
Frequent sharing is childish	

Q8. The factors that motivate you to express and share your life on Weibo include:

* 【Multiple choice questions】

Motivating Factors<br>Tick	√
Positive feedback from friends	
Get social support	
Contribute to the advancement of work	
Helps manage social relationships	
Have the desire to share	
Emotions are rich and need to be vented	
Help others understand me	
Spreading useful information to others	

Q9. To what extent do you believe that you can judge the true condition of others through Weibo information?

* [Slider question]

Not at all, basically not, sometimes, often, completely

1 2 3 4 5

Q10. What is the relationship between the self-presented in the content you post on Weibo and the real self?

* [Slider question]

Not at all,	basically not,	sometimes,	often,	completely
1	2	3	4	5

Q11. Can people judge the real you from the information in Weibo?

* [Slider question]

Not at all,	basically not,	sometimes,	often,	completely
1	2	3	4	5

Q12. To what extent do you agree with the following statement?

* [Matrix Questions]

	**Absolutely Disagre**				**Absolutely Agree**
I think images and video footage express themselves better than text.	1	2	3	4	5
When I express my opinions on Weibo, I will not be thoughtful, and will not use every word carefully.	1	2	3	4	5
The me on Weibo is the real me.	1	2	3	4	5
The me on Weibo is the complete me.	1	2	3	4	5
I often post unretouched (no p-picture, beauty camera) photos on Weibo.	1	2	3	4	5
The Internet image in Weibo will have an impact on my real life.	1	2	3	4	5
I have a clear willingness to maintain a good online image on Weibo.	1	2	3	4	5

I know what my ideal online image on Weibo looks like.	1	2	3	4	5

Q13. What is your gender?

Male

Female

Q14. What is your age?

Under 18

18-24 years old

25-29 years old

30-34 years old

35-39 years old

40-44 years old

45-49 years old

50-54 years old

55-59 years old

Over 60

Q15. What is your current highest education?

Elementary school and below

Junior high school

High school/secondary school/technical school/vocational high school

College

Undergraduate

Master's degree and above

Q16. What is your average monthly disposable personal income?

(Reminder: Refers to the total after-tax income including wages, bonuses, allowances, etc., after deducting fixed expenses such as housing loan and car loan, and apportion it to monthly income)

Below 500 yuan

501-1000 yuan

1001-2000 yuan

2001-3000 yuan

3001-5000 yuan

5001-8000 yuan

8001-10000 yuan

10001-15000 yuan

15001-20000 yuan

More than 20001 yuan

Q17. What is your current province, autonomous region or municipality?

34 provincial drop-down lists, prefecture-level drop-down lists

Q18. Where is your current residence?

Prefectural-level municipal districts

County-level cities and surrounding counties of prefecture-level cities

Township

rural
